# Does [99mTc]-3,3-diphosphono-1,2-propanodicarboxylic acid (DPD) soft tissue uptake allow the identification of patients with the diagnosis of cardiac transthyretin-related (ATTR) amyloidosis with higher risk for polyneuropathy?

**DOI:** 10.1007/s12350-022-02986-7

**Published:** 2022-07-11

**Authors:** Tim Wollenweber, Elisabeth Kretschmer-Chott, Raphael Wurm, Sazan Rasul, Oana Kulterer, Rene Rettl, Franz Duca, Diana Bonderman, Kurt-Wolfram Sühs, Marcus Hacker, Tatjana Traub-Weidinger

**Affiliations:** 1 Division of Nuclear Medicine, Department of Biomedical Imaging and Image-Guided Therapy, Medical University of Vienna, Vienna, Austria; 2 Department of Neurology, Medical University of Vienna, Vienna, Austria; 3 Division of Cardiology, Department of Internal Medicine II, Medical University of Vienna, Vienna, Austria; 4 5th Medical Department with Cardiology, Clinic Favoriten, Vienna, Austria; 5 Department of Neurology, Hannover Medical School, Hannover, Germany

**Keywords:** Amyloidosis, ATTR, Bone scintigraphy, Polyneuropathy

## Abstract

**Background:**

With the introduction of several drugs for the therapy of transthyretin-related amyloidosis (ATTR) which slow down the disease, early detection of polyneuropathy (PNP) is becoming increasingly of interest. [99mTc]-3,3-Diphosphono-1,2-Propanodicarboxylic Acid (DPD) bone scintigraphy, which is used for the diagnosis of cardiac (c)ATTR, can possibly make an important contribution in the identification of patients at risk for PNP.

**Methods:**

Fifty patients with cATTR, who underwent both planar whole-body DPD scintigraphy and nerve conduction studies (NCS) were retrospectively evaluated. A subgroup of 22 patients also underwent quantitative SPECT/CT of the thorax from which Standardized Uptake Values (SUVpeak) in the subcutaneous fat tissue of the left axillar region were evaluated.

**Results:**

The Perugini score was significantly increased in patients with cATTR and additional diagnosis of PNP compared to patients without (2.51 ± 0.51 vs 2.13 ± 0.52; *P* = 0.03). Quantitative SPECT/CT revealed that DPD uptake in the subcutaneous fat of the left axillar region was significantly increased in cATTR patients with compared to patients without (1.36 ± 0.60 vs 0.74 ± 0.52; *P* = 0.04).

**Conclusion:**

This study suggests that DPD bone scintigraphy is a useful tool for identification of patients with cATTR and a risk for PNP due to increased DPD soft tissue uptake.

**Supplementary Information:**

The online version contains supplementary material available at 10.1007/s12350-022-02986-7.

## Introduction

Systemic amyloidosis is a group of diseases with the accumulation of misfolded proteins mostly extracellularly in the interstitium of different organs, finally resulting in a loss of normal tissue architecture and function.^1^ The most common forms of amyloidosis include light chain (AL), Amyloid A (AA), and Transthyretin-related (ATTR) amyloidosis. TTR, which is a transport protein for thyroxine and vitamin A,^2^, is synthesized in the liver, choroid plexus, and retina.^3^ The destabilization of the homeotetrameric structure of this protein leads to a disassociation into TTR monomers preceding amyloid fibril formation causing ATTR.^4^ ATTR can be present in two forms, the acquired wild-type (wt)ATTR, which is associated with a misaggregation of wild-type transthyretin, and the hereditary form, also known as hATTR, which is associated with a misfolding of transthyretin due to mutations in the corresponding transthyretin gene. To date, over 100 different mutations of the TTR gene have been described.^5^ The deposition of the amyloid fibrils in hATTR occurs in the heart, peripheral nerves, and other organs, which may cause axonal sensory autonomic and motor neuropathy such as in (familial amyloid polyneuropathy; FAP).^2,6^ Although peripheral or autonomic neuropathy is regarded as less common in patients with wtATTR than in hATTR,^2,7–9^ it also occurs in a certain proportion of them.^10^

Moreover, post-mortem studies were able to demonstrate TTR amyloid deposition regardless of the type of ATTR in almost all organs and tissues of affected persons^11–14^ Therefore, patients with ATTR may show different patterns of neuropathy. Electroneurophysiological studies with needle EMG, motor conduction velocities, sensory conduction velocities, sensory potentials, and nerve potentials can be useful to determine the involvement of peripheral nerves and may detect the disease before it is clinically obvious.^15^

In cardiac (c)ATTR nuclear whole-body (WB) imaging with radiolabeled phosphonates such as [99mTc]-9mTc-3,3-Diphosphono-1,2-Propanodicarboxylic acid (DPD) have shown a strong affinity for TTR amyloid fibril infiltrations in the heart and is very effective in detecting cATTR.^16–19^ Up to date the exact mechanism of binding is unclear, although the calcium content of TTR may play an important role.^20,21^ Moreover, a DPD uptake of radiolabeled phosphonates in cATTR patients has been also observed in other organs, like the lung or the spleen.^22,23^ For describing the tracer distribution, Perugini et al^16^ developed a four-point visual scoring system depending on the cardiac uptake, the attenuation of bone uptake, and also corresponding to a soft tissue (ST) uptake, which has been clinically established over the last years. According to the recently published ASNC guidelines on cardiac amyloid imaging, bone scintigraphy with DPD, hydroxy-dimethylene diphosphonate (HMDP), or Pyrophosphate (PYP) has become part of the algorithm for evaluating patients with cardiac amyloidosis.^24^

Depending on the type of ATTR amyloidosis DPD bone scintigraphy may show a different ST uptake pattern in patients with cATTR. In particular, patients with wtATTR and hATTR-V122I amyloidosis have been recently described with higher diffuse skeletal and muscle uptake compared to patients with other mutations.^22^ Therefore, little is known regarding the ST tracer uptake behavior in patients with cATTR and their possible relevance in terms of neurological diseases. Hence, we hypothesize that a higher DPD ST uptake will be observed in patients with known polyneuropathy using the visual Perugini score (PS) as well as quantitative parameters of planar and SPECT/CT DPD scintigraphy.

## Methods

### Patients

Fifty patients with cATTR (41x WT, 9x hATTR: 1x Thr69Ile, 5x His108Arg, 1x Thr80Ala, 1xVal50Met, 1x Val113Leu) were included into this retrospective study. The group consisted of 40 men and 10 women with a mean age of the 77.4 ± 8.2 years. Appropriate ethical approval was obtained by the ethics committee of the Medical University of Vienna (No: 1443/2021). In 36 of these patients the diagnosis of cATTR was established based on Perugini grade 2 or 3 and the absence of a monoclonal protein in blood and urine^19^ and in 12 patients with endocardial biopsy. Two more patients showed positive DPD scans and positive genetic testing. Moreover, 9 out of the 50 patients had an additional history of diabetes. All patients had routinely a performed nerve conduction study (NCS) with a mean time interval between bone scan and NCS of 210 ± 93 days. The NVC measurements revealed 25 patients with presentation of sensorimotor polyneuropathy (19x wtATTR, 6x hATTR) and 10 patients with presentation of a sensory polyneuropathy (9x wtATTR, 1x hATTR [His108Arg]). The group of the remaining 15 patients did not show any sign of polyneuropathy (13 x wtATTR, 2 x hATTR). See also Table 1.

**Table 1:** Patient population

	n	Mean Age	Gender (m/w)	PNP (n)	No PNP (n)	Diabetes mellitus (n)
wtATTR	41	76.6 ± 12.5	34/7	28	13	8 (6x PNP vs 2x No PNP)
hATTR	9	65 ± 5.7	6/3	7 (1x Thr69Ile, 1x Thr80Ala, 4x His108Arg, 1x Val50Met)	2 (His108Arg, Val113Leu)	1 (1x No PNP vs 0x PNP)

### Imaging and data analysis

All patients were imaged with a DPD bone scintigraphy at the Division of Clinical Nuclear Medicine of the Medical University of Vienna. Planar WB images were obtained 2.5 h after intravenous injection of 700 MBq [99mTc]-radiolabeled DPD. Forty-eight patients underwent planar imaging on a hybrid SPECT/CT system Symbia Intevo (Siemens Medical Solutions AG, Erlangen, Germany) and 5 patients on a double-headed gamma camera Axis (Philips Medical Systems, Amsterdam, the Netherlands). In a subgroup of 22 patients SPECT/CT of the thorax was performed direct after planar imaging using the Siemens Symbia Intevo SPECT/CT system equipped with a low-energy high-resolution collimator. Images were acquired in 180° configuration, 64 views, 20 s per view, 256 × 256 matrix, and an energy window of 15% around the 99mTc photopeak of 141 keV. Subsequent to the SPECT acquisition, a low-dose CT scan was done for attenuation correction (130 kV, 35 mAs, 256 ×256 matrix, step-and-shoot acquisition with body contour). For image acquisition and reconstruction xSPECT/CT QUANT technology was utilized. See also Table 2 for description of the study population.

**Table 2:** Patients with additional quantitative thoracic SPECT/CT

	n	Mean age	Gender (m/w)	PNP (n)	No PNP (n)	Diabetes mellitus (n)
wtATTR	14	81.7 ± 4.8	12/2	9	5	2 (1x No PNP vs 1x PNP)
hATTR	8	65.5 ± 5.8	5/3	6 (4x His108Arg, 1x Thr80Ala, 1x Val50Met)	2 (His108Arg, Val113Leu)	1 (0x No PNP vs 1x PNP)

**Figure 1:**
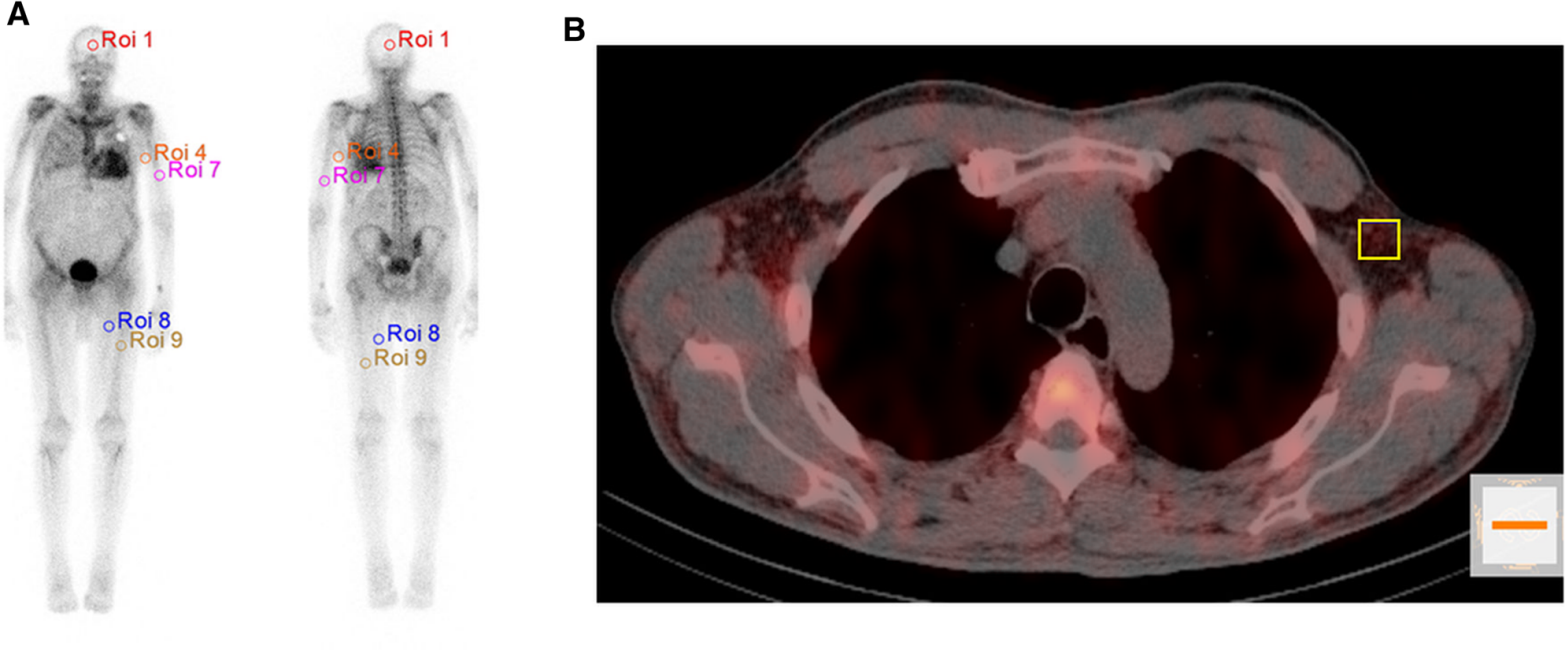
Evaluation methodology of DPD bone scintigraphy for cardiac amyloidosis. (**a**) Showing the ROI localization for the skull uptake (red ROI1) as well as the ratio of soft tissue to humeral shaft (orange ROI 4 and pink ROI 7) and the soft tissue to femoral shaft estimation (blue ROI 8 and brown ROI 9) and (**b**) for the soft tissue tracer uptake of subcutaneous fat in the left axillar region

For visual image analysis planar WB images were evaluated by two experienced physicians independently and visually graded according to the PS (i.e., score 0, absent cardiac uptake and normal bone uptake; score 1, mild cardiac uptake, inferior to bone uptake; score 2, moderate cardiac uptake accompanied by attenuated bone uptake; score 3, strong cardiac uptake with mild/absent bone uptake).^16^ Grading discrepancies were again discussed and resolved by consensus. For quantitative measurements the WB DPD uptake was calculated as geometric mean of anterior and posterior counts of a WB ROI (region of interest) divided by the applied activity. ST uptake on planar images was estimated by placing a 3-cm circular ROI on the inside of the left proximal upper and lower limb and then calculating it as the geometric mean of the anterior and posterior counts normalized to applied activity. Similar procedures were performed for the left humerus and left femoral bone shaft as well as for the skull (for better illustration see also Fig. 1a.) In a subgroup of patients, SPECT/CT image data generated with the commercially available Hermes Hybrid 3D software package (Hermes Medical Solutions, Stockholm, Sweden) were used to calculate DPD uptake in ST. Because SPECT/CT image acquisition was primarily performed over the thorax, the subcutaneous abdominal region was not available for evaluation in all patients. Therefore, a 2.92-ml cubic volume of interest (VOI) was placed in the subcutaneous fat of the left axillary region (see also Fig. 1b), from which uptake was determined as SUVpeak.

**Figure 2:**
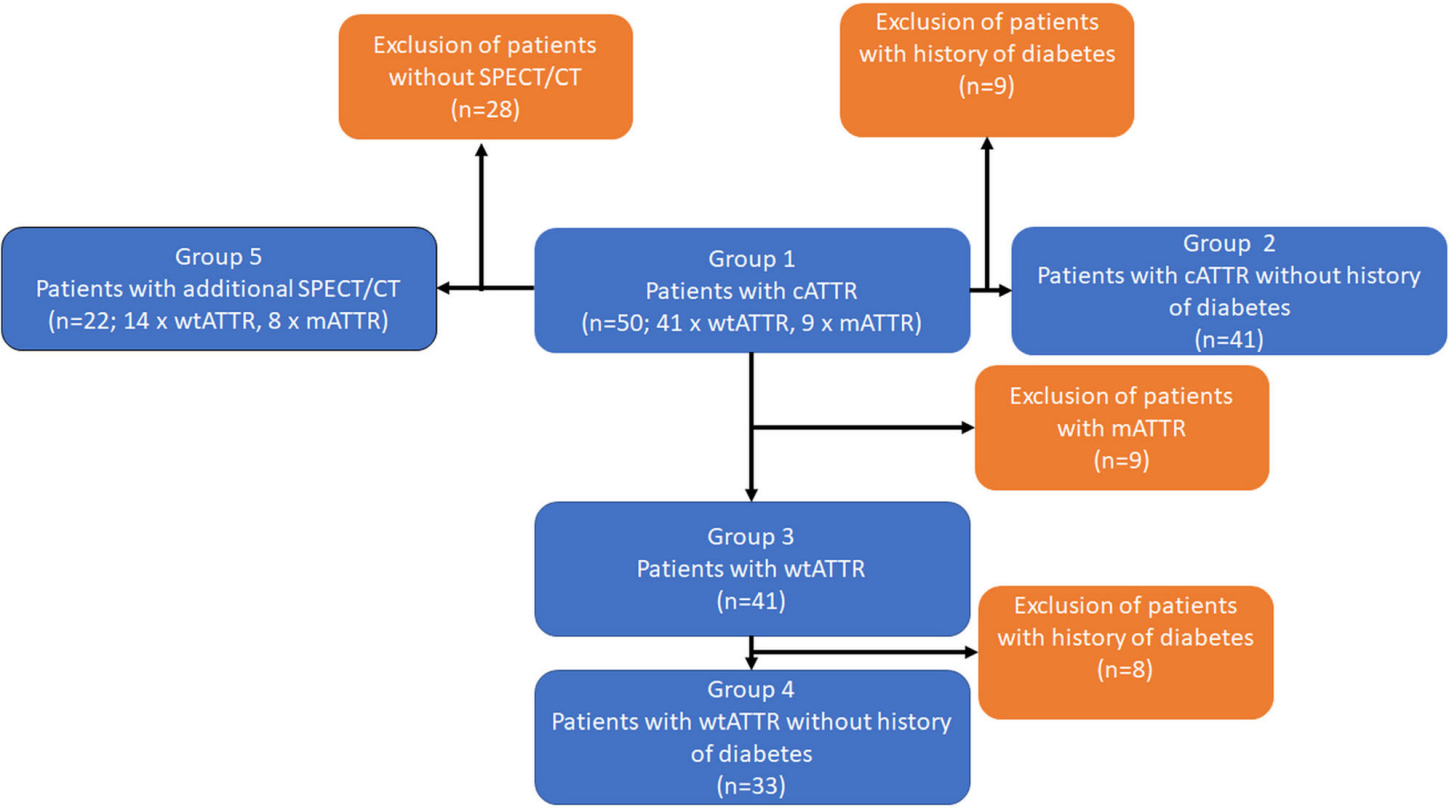
Diagram illustrating the division of patients discussed in the methods for further data analysis. This diagram illustrates the classification of patients discussed in methods for further data analysis

For further data analysis patients were divided into following groups (see also Fig. 2):

*Group 1*: all 50 patients.

*Group 2*: patients of group 1 with exclusion of patients with diabetes mellitus.^25,26^

*Group 3*: patients with only wtATTR.

*Group 4*: patients with only wtATTR after exclusion of patients with diabetes mellitus.

*Group 5*: patients with additional chest SPECT/CT.

### Statistics

Statistical analysis was performed using MedCalc v19.1 (Ostend, Belgium). Linear regression analysis was used to describe the relationship between continuous variables. The Kolmogorov–Smirnov test was performed to check for normal distribution. For normally distributed data comparisons between groups with polyneuropathy and without polyneuropathy in NCS was performed using the student’s t test. If the data were not normally distributed the Wilcoxon rank sum test is used for the comparison between both groups. Non-normally distributed data were expressed as medians and ranges. For the comparison of ordinally scaled data the Wilcoxon rank sum test was used. Ordinally scaled data were presented as mean and standard deviation. One-way ANOVA was used to evaluate whether age differences exist between patients with different PS. As a post hoc test, the Scheffé test was conducted to find out which pairs of means were significant. A value of *P* <0.05 was considered statistically significant.

## Results

### Visual DPD bone scintigraphy analysis

Among the 41 patients with wtATTR, 1 patient was rated as Perugini grade 1, 27 patients as Perugini grade 2, and 13 as Perugini grade 3. Regarding the 9 patients with hATTR, 1 patient was rated as Perugini grade 2 (Val113Leu) and 8 patients as Perugini grade 3 (1x Thr69Ile, 5x His108Arg, 1x Thr80Ala, 1x Val50Met). WB imaging analysis of all 50 patients showed a significantly higher Perugini grading of cATTR patients with PNP compared to patients without (2.51 ± 0.51 vs 2.13 ± 0.52; *P* = 0.03). In group 2, reflecting only cATTR patients without a history of diabetes mellitus, the results became even more significant (2.62 ± 0.49 vs 2.08 ± 0.29; *P* = 0.001). See also Table 3. The analysis of 41 patients with wtATTR (group 3) only revealed a trend of higher Perugini grading in patients with compared to patients without PNP (2.39 ± 0.50 vs 2.08 ± 0.49; *P* = 0.08). However, after exclusion of patients with diabetes mellitus (group 4) the difference became significant (2.50 ± 0.51 vs 2.09 ± 0.30; *P* = 0.02). See also Table 4.

**Table 3:** Results for cardiac amyloidosis patients with and without PNP regarding visual Perugini score and quantitative whole-body and skull DPD uptake as well as femur to soft tissue and humerus to soft tissue ratios

	Group 1 – cATTR	Group 2- cATTR without DM
Perugini score PNP vs no PNP	(2.51 ± 0.51 vs 2.13 ± 0.52; *P* = 0.03) (N = 50; 35 vs 15 pat.)	(2.62 ± 0.49 vs 2.08 ± 0.29; *P* = 0.001) (N = 41; 29 vs 12 pat.)
Whole-body Uptake [cts/MBq] PNP vs no PNP	1928 ± 291 vs 1867 ± 272; *P* = 0.51 (N = 45; 31 vs 14 pat.)	1926 ± 309 vs 1875 ± 247; *P* = 0.63 (N = 36; 25 vs 11 pat.)
Skull uptake PNP vs no PNP [cts/MBq]	1.24 ± 0.44 vs 1.68 ± 0.63; *P* = 0.01 (N = 45; 31 vs 14 pat.)	1.20 ± 0.46 vs 1.76 ± 0.62; *P* = 0.01 (N = 36; 25 vs 11 pat.)
Soft tissue/Femoral shaft PNP vs no PNP	0.96 ± 0.14 vs 0.89 ± 0.16; *P* = 0.11 (N = 45; 31 vs 14 pat.)	0.97 ± 0.14 vs 0.88 ± 0.12; *P* = 0.07 (N = 36; 25 vs 11 pat.)
Soft tissue/humeral shaft PNP vs no PNP	0.91 ± 0.36 vs 0.83 ± 0.24; *P* = 0.49 (N = 45; 31 vs 14 pat.)	0.94 ± 0.39 vs 0.83 ± 0.26; *P* = 0.37 (N = 36; 25 vs 11 pat.)

**Table 4:** Results of 41 wtATTR patients with and without PNP regarding visual Perugini grading and quantitative bone DPD uptake

	Perugini PNP vs no PNP	Skull uptake* PNP vs no PNP
wtATTR (41 pat)	(2.39 ± 0.50 vs 2.08 ± 0.49; *P* = 0.08) 28 vs 13 pat	1.27 ± 0.43 vs 1.65 ± 0.58; *P* = 0.03 23 vs 13 pat
wtATTR without DM (33)	(2.50 ± 0.51vs2.09 ± 0.30; *P* = 0.02) 22 vs 11 pat	1.22 ± 0.46 vs 1.69 ± 0.54; *P* = 0.02 17 vs 11 pat

### Quantitative DPD bone scintigraphy analysis

In order to ensure comparability of quantitative data of WB imaging and ROI-based skeletal and ST measurements, only patients examined on the Siemens Symbia Intevo camera system were included in the further analysis (n = 45). Regarding whole-body DPD uptake normalized to the applied activity there was no significant difference between patients of group 1 with compared to those without PNP (1928 ± 291 cts/MBq vs 1867 ± 272 cts/MBq; *P* = 0.51). Even after exclusion of patients with diabetes (group 2) no further significant differences were observed (1926 ± 309 cts/MBq vs 1875 ± 247 cts/MBq; *P* = 0.63). Moreover, ROI analysis revealed that skull uptake was significantly decreased in patients of group 1 with compared to without PNP (1.24 ± 0.44 cts/MBq vs 1.68 ± 0.63 cts/MBq; *P* = 0.01), which also remained significant in group 2 (1.20 ± 0.46 cts/MBq vs 1.76 ± 0.62 cts/MBq; *P* = 0.01; Online Resource Fig. ESM1). Detailed results of skull and other bone regions for group 1 and 2 are shown in Table 3. In patients with only wtATTR (group 3), skull uptake was significantly decreased in patients with PNP compared to patients without (1.27 ± 0.43 cts/MBq vs 1.65 ± 0.58; *P* = 0.03). Even after exclusion of patients with diabetes (group 4) the skull uptake in wtATTR patients with PNP remained significantly decreased (1.22 ± 0.46 cts/MBq vs 1.69 ± 0.54 cts/MBq; *P* = 0.02; Online Resource Fig. ESM2 a, b, Table 4). Additional ST-based ratio analysis of group 1 did not reveal any significant differences (Table 3).

**Figure 3:**
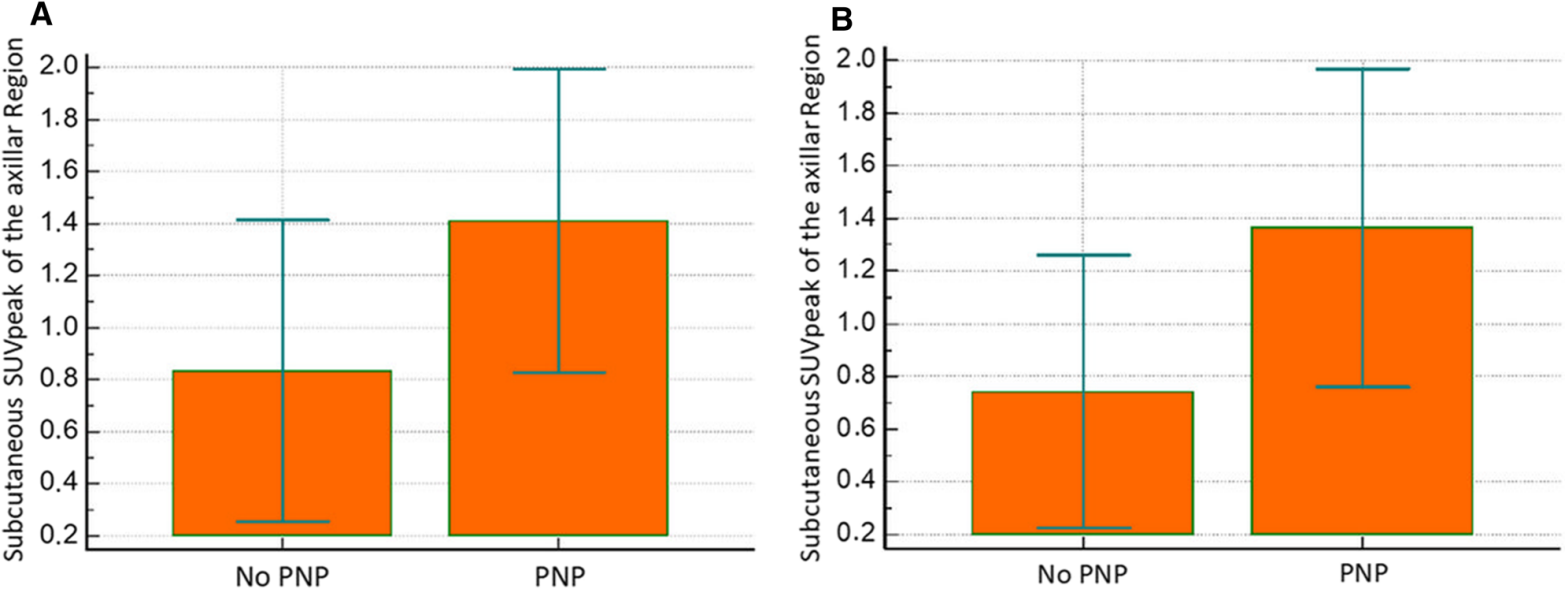
Comparison of the soft tissue tracer uptake of subcutaneous fat in the left axillar region in patients with vs without PNP using DPD SPECT/CT scintigraphy. (**a**) In the subgroup of the 22 patients with quantitative thoracic SPECT/CT the soft tissue tracer uptake of subcutaneous fat in the left axillar region was significantly increased in patients with PNP compared to patients without PNP (1.41 ± 0.58 vs 0.84 ± 0.58; *P* = 0.04). (**b**) After exclusion of patients with diabetes the soft tissue tracer uptake of subcutaneous fat in the left axillar region was still significantly increased in patients with PNP compared to patients without PNP (1.36 ± 0.60 vs 0.74 ± 0.52; *P* = 0.04)

The thoracic SPECT/CT subgroup analysis (group 5) showed a significantly increased DPD uptake of the subcutaneous fat in the left axillar region in cATTR patients with PNP compared to patients without (1.41 ± 0.58 vs 0.84 ± 0.58; *P* = 0.04). After excluding patients with diabetes ST uptake further increased significantly (1.36 ± 0.60 vs 0.74 ± 0.52; *P* = 0.04, Fig. 3). Representative SPECT/CT images of hATTR and wtATTR patients with and without PNP are shown in Online Resource Fig. ESM3. The additional SPECT/CT subgroup analysis of 14 wtATTR patients did not reveal any significant difference of SUVpeak in the subcutaneous fat of the left axillar region in patients with compared to patients without PNP (1.19 ± 0.65 vs 0.53 ± 0.37; *P* = 0.06), even after exclusion of patients with diabetes (1.17 ± 0.66 vs 0.53 ± 0.37; *P* = 0.08, Online Resource Table ESM1).

## Discussion

With the introduction of several drugs for the therapy of ATTR, which slow down disease progression also in patients with ATTR-related polyneuropathy^27,28^ and improve the quality of life,^29,30^ early detection of this form of PNP is increasingly important for treatment decision. However, early detection of such patients is hampered by the diversity of clinical features in ATTR amyloidosis at the time of diagnosis and in course of the disease.^31^

**Figure 4:**
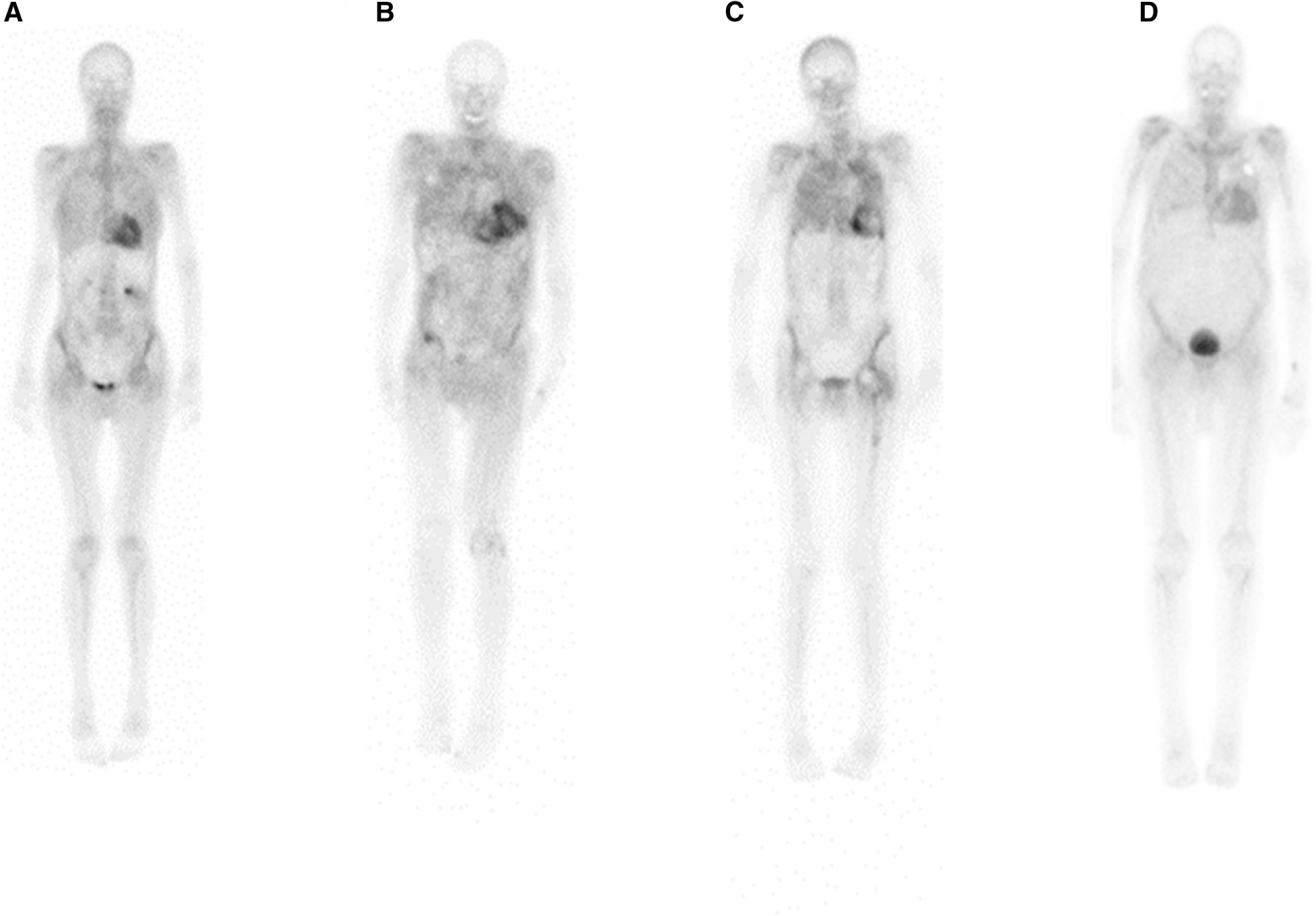
Representative examples for planar DPD bone scintigraphy of hATTR and wtATTR patients with and without PNP (with windowing of an upper threshold of 100% of the maximum). (**a**) a hATTR patient (his108arg) with PNP revealing a generally increased soft tissue uptake (Perugini score 3), (**b**) a wtATTR patient with PNP showing nearly absent bone uptake (Perugini score 3), (**c**) a hATTR patient (Val113Leu) without PNP with low-attenuated bone uptake (Perugini score 2), especially in the region of the femoral shaft, and (**d**) a wtATTR patient without PNP who also shows also only low-attenuated bone uptake (Perugini score 2)

By comparing cATTR patients with PNP based on NCS measurements to those without PNP, we demonstrated that PS was significantly increased in patients with PNP. This observation indicates that patients with higher Perugini grades reflect a possible PNP involvement, especially patients with Perugini grade 3. Interestingly, after excluding patients with diabetes mellitus, to avoid patients having PNP due to diabetes mellitus and not due to ATTR, the difference became more significant in our study. The main difference between PS 2 and 3 is the mild or absent bone uptake observed in grade 3, which is caused by an increased ST uptake. Fig. 4 illustrates this observation. These findings are also supported and confirmed with a 3D SPECT/CT analysis of 22 patients with cATTR showing that the soft tissue uptake in the left axillar region after exclusion of patients with diabetes mellitus was significantly increased in patients with PNP compared to patients without.

Moreover, the fact that the activity in the skull was significantly decreased in cATTR patients with PNP compared to patients without is consistent with an increased ST uptake in this patient population. This is surprising but may be explained as a competition of amyloid deposits ready for radiotracer uptake between myocardial, bone, and especially the ST rather than only reflecting bone activity, also discussed by other groups.^32–34^ Our hypothesis that a generalized increased ST DPD uptake in cATTR patients is related to PNP is also supported by Takahashi et al, discussing that in patients with cATTR abnormal PYP uptake in the subcutaneous abdominal fat could reflect the regional amyloid deposition. Rokke et al observed that patients with merely neuropathic symptoms display higher sensitivity for the diagnosis of hereditary ATTR based on abdominal fat pad biopsies compared to patients with pure cardiomyopathy (CM).^35,36^ Interestingly, in our analysis of wtATTR patients with additional quantitative SPECT/CT SUVpeak of the subcutaneous fat showed only a trend to be significantly increased in patients with PNP. It may be noticeable that these results appear somewhat worse than for the entire group with hATTR patients combined. Hutt et al^22^ showed particularly in patients wtATTR and hATTR-V122I amyloidosis a predominantly muscular uptake pattern involving the gluteal, shoulder, chest, and abdominal wall regions. These observations and the fact, that in patients with wtATTR bilateral carpal tunnel syndrome, biceps tendon rupture, and spinal stenosis are more common clinical symptoms than PNP,^2,7,8^ may indicate that amyloid deposition in subcutaneous fat tissue, which then is probably accompanied by increased amyloid deposition in nerve fibers, rather than in muscles seems to be the cause of PNP. This hypothesis is also supported by the observation of decreased subcutaneous fat DPD uptake in wtATTR patients compared to hATTR (Thr80Ala, His108Arg, Val50Met, Val113Leu)^33^ and by a relatively low sensitivity of abdominal fat biopsy in wATTR patients compared to hATTR patients.^37,38^ In a previous study with PYP only a minimal skeletal muscle uptake of PYP was observed in patients with cATTR amyloidosis and no relation to PNP.^39,40^ Therefore, extracardiac uptake quantification is a potential benefit of DPD over PYP.

In our subgroup analysis of patients with only wtATTR we also found a significantly reduced tracer uptake in the skull of patients with compared to patients without PNP in planar imaging, prior and after excluding patients with diabetes mellitus, and additionally a significant higher PS in patients without diabetes mellitus. Therefore, DPD bone scintigraphy may be also useful in patients with cardiac wtATTR for evaluation regarding an involvement of PNP.

A further observation of the presented study is that the ST analysis using planar imaging data of upper and lower extremities did not reveal a significant difference between patients with compared to patients without PNP also after exclusion of patients with diabetes mellitus. This is probably due to the high variability of the measured counts caused by a small change of the position of the ST ROI and therefore this may not be the right procedure for identification of patients with increased risk of PNP. Nevertheless, planar WB showed no significant difference of WB uptake between cATTR patients with compared to patient without PNP. This reflects even more a different distribution pattern of the radiopharmaceutical in the various compartments (myocardium, muscle or subcutaneous fat, bone) than an overall increased tracer uptake in patients with cardiac amyloidosis and PNP compared to patients without PNP.

To better understand our results, we can only speculate how the different tracer distribution pattern in the adipose tissue instead of muscular tracer uptake leads to PNP. The increased tracer accumulation as imaging correspondent to amyloid deposition in the adipose tissue may be also associated to amyloid deposition in the peripheral nerve running in the same region, damaging the nerves by several known mechanisms, such as mechanical compression, angiopathy with direct blood vessel invasion, enhanced leakage of circulating TTR, and toxicity of nonfibrillar TTR.^41,42^ Amyloid accumulations in ST, particularly in subcutaneous adipose tissue, may also increase over time and therefore simply reflect a more advanced stage of disease. A further possible explanation may be that recently two different types of amyloid fibrils have been identified. The amyloid fibril composition determines the clinical phenotype of disease. While patients with type A fibrils present a mixture of neuropathy and CM, patients with type B fibrils are described with neuropathy at onset.^43,44^ However, since type B fibrils seem to show no DPD uptake in bone scintigraphy,^45,46^ it is very unlikely that the increased DPD uptake in ST in patients with PNP is caused by “neuropathic” type B fibrils.

## Study Limitations

Some limitations of the present study need to be recognized. One limitation is the relatively small sample size of subjects which is not unusual for such a rare disease. Furthermore, this is a retrospective study and the relative long time between bone scintigraphic imaging and NCS measurements and the lack of information regarding the onset of PNP may lead to the bias that patients evaluated in different stages of disease, although significant imaging differences were observed for cATTR patients with compared to without PNP. Another limitation is the limited knowledge about the kidney function of the patients. However, since the creatinine levels between patients with and without PNP showed no significant difference regarding PNP (1.29 ± 0.39 vs 1.18 ± 0.33; *P* = 0.34), it seems to be more unlikely that the increased ST DPD accumulation in patients with PNP was caused by an advanced renal disease. There are several studies with PYP and HMDP, which outlined how quantitative measures correlate with cardiac disease burden^47–50^. On the basis of these studies, it appears possible that patients with a higher PS or ST accumulation are in a more advanced stage of disease. However, one-way ANOVA revealed significant differences between patients with PS 1, 2, and 3 (*P* = 0.004), where Scheffé test as a post hoc test revealed that patients with PS 3 were significantly younger than patients with PS 2 (80.5 ± 6.2 vs 72.9 ± 9.3 years; *P* < 0.05). Another fact that speaks against this assumption is that there was no significant age difference between patients with and without PNP (77.6 ± 8.6 vs 76.7 ± 7.4 years, *P* = 0.70). These two facts make it less likely that the increased ST accumulation can be simply explained by an only prolonged course of the disease. Furthermore a previous study^33^ observed that patients with hATTR show a higher ST uptake than those with wtATTR; this suggests that at least the stage of the disease cannot be the only cause for the results of the study. Nevertheless, since this is a retrospective study, it cannot be excluded that some of the findings are associated with a more advanced disease. A further limitation is that we only analyzed patients with or without diabetes mellitus, but other factors such as chemotherapy or increased alcohol consumption may also influence the development of PNP. As far as we could figure out from the clinical information system, none of the patients had a previous chemotherapy, increased alcohol consumption was not documented. Finally, regional heterogeneity of amyloidosis genotype and phenotype across Europe is well known and may also influence the results of the study.^51^ In Austria 10 different TTR missense mutations (p.Val40Ile, p.Arg41Gln, p.Val50Met, p.Thr69Ile, p.Thr80Ala, p.His108Arg, p.Val113Leu, p.Val114Ala, p.Ile127Phe, p.Val142Ile) have been detected yet and therefore or data may be not reproducible in regions with a different genetic spectrum compared to Austria.^52^ Finally, since a Bonferroni correction was not performed due to the sample size of the study, we cannot exclude a potential increase in type 1 errors that may have influenced the study results.

## New Knowledge Gained

DPD bone scintigraphy does not give only information regarding possible cardiac involvement in patients with ATTR but also allows identification of patients with potential risk for PNP based on increased soft tissue DPD uptake.

## Conclusion

In conclusion, DPD bone scintigraphy gives more information than only a possible cardiac involvement in patients with ATTR. The results of our retrospective study suggest that it may be possible to identify patients at risk for PNP based on increased ST uptake, particularly in subcutaneous adipose tissue. In addition, quantitative SPECT/CT methods offered a clear advantage over the examiner-dependent visual Perugini scoring system. Nevertheless, further studies with a broader population spectrum and a prospective study design should be conducted to confirm these observations.

## Supplementary Information

Below is the link to the electronic supplementary material.

Electronic supplementary material 1 (PPTX 754 kb)

Electronic supplementary material 2 (DOCX 12 kb)

Electronic supplementary material 3 (TIF 432 kb)

Electronic supplementary material 4 (DOCX 13 kb)

Electronic supplementary material 5 (TIF 568 kb)

Electronic supplementary material 6 (DOCX 12 kb)

Electronic supplementary material 7 (TIF 544 kb)

## Abbreviations

HMPD: Hydroxy-dimethylene diphosphonate
WB: Whole body
PS: Perugini score
ST: Soft tissue
CM: Cardiomyopathy
ATTR: Transthyretin-related amyloidosis
wt: Wild-type
(h)ATTR: Hereditary
PNP: Polyneuropathy
DPD: 3,3-Diphosphono-1,2-propanodicarboxylic acid
