# Supplementary material for: Does [99mTc]-3,3-diphosphono-1,2-propanodicarboxylic acid (DPD) soft tissue uptake allow the identification of patients with the diagnosis of cardiac transthyretin-related (ATTR) amyloidosis with higher risk for polyneuropathy?
Source: J Nucl Cardiol. 2022 Jul 11;30(1):357–67. doi: 10.1007/s12350-022-02986-7 (PMC9984356; doi:10.1007/s12350-022-02986-7)
Supplement: Supplementary file 1 — Electronic supplementary material 1 (PPTX 754 kb) [file 12350_2022_2986_MOESM1_ESM.pptx]

## Slide 1
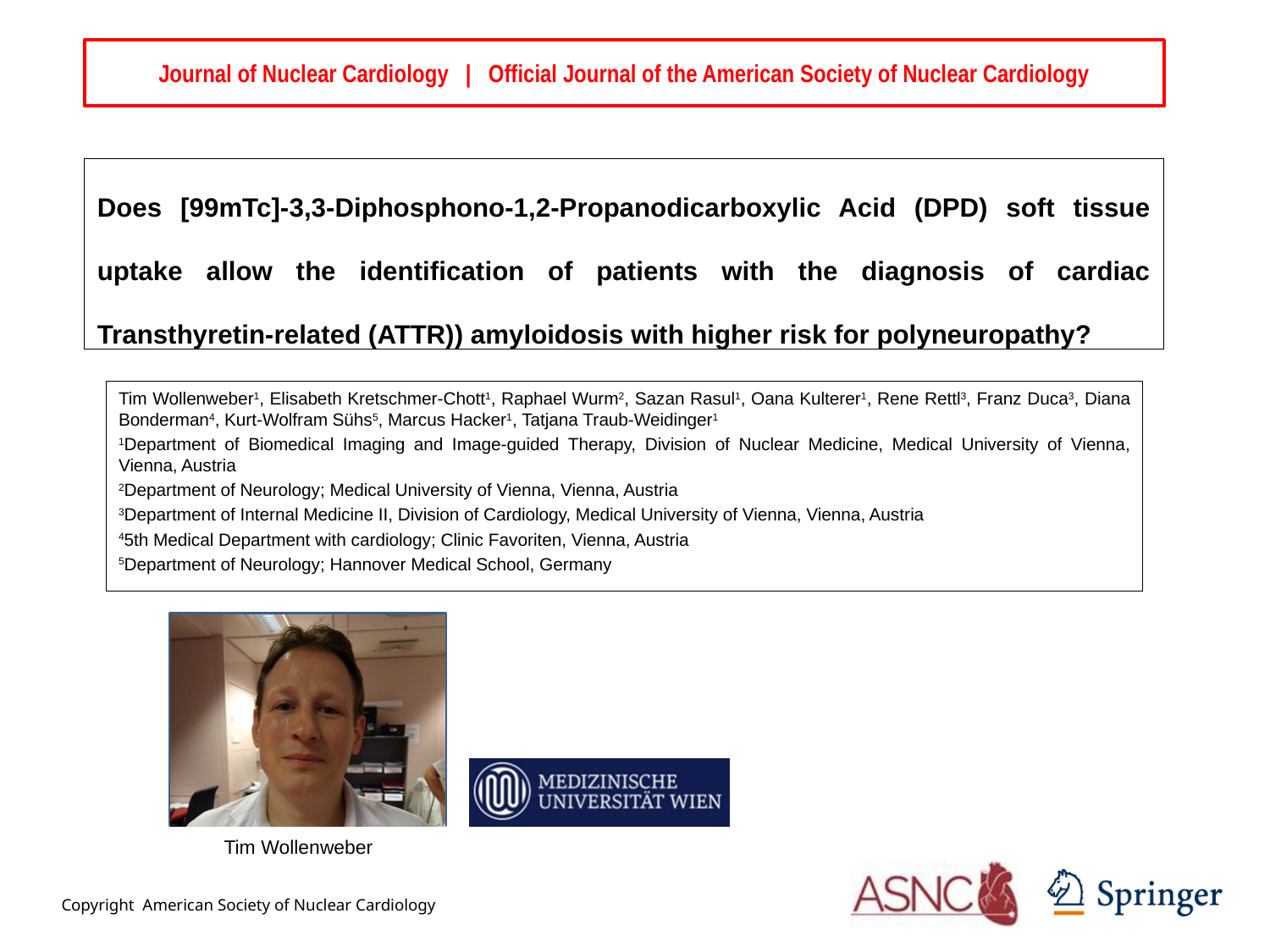

Journal of Nuclear Cardiology | Official Journal of the American Society of Nuclear Cardiology
# Does [99mTc]-3,3-Diphosphono-1,2-Propanodicarboxylic Acid (DPD) soft tissue uptake allow the identification of patients with the diagnosis of cardiac Transthyretin-related (ATTR)) amyloidosis with higher risk for polyneuropathy?
Tim Wollenweber1, Elisabeth Kretschmer-Chott1, Raphael Wurm2, Sazan Rasul1, Oana Kulterer1, Rene Rettl3, Franz Duca3, Diana Bonderman4, Kurt-Wolfram Sühs5, Marcus Hacker1, Tatjana Traub-Weidinger1
1Department of Biomedical Imaging and Image-guided Therapy, Division of Nuclear Medicine, Medical University of Vienna, Vienna, Austria
2Department of Neurology; Medical University of Vienna, Vienna, Austria
3Department of Internal Medicine II, Division of Cardiology, Medical University of Vienna, Vienna, Austria
45th Medical Department with cardiology; Clinic Favoriten, Vienna, Austria
5Department of Neurology; Hannover Medical School, Germany
Head shot of author
required
Tim Wollenweber
Copyright American Society of Nuclear Cardiology

## Slide 2
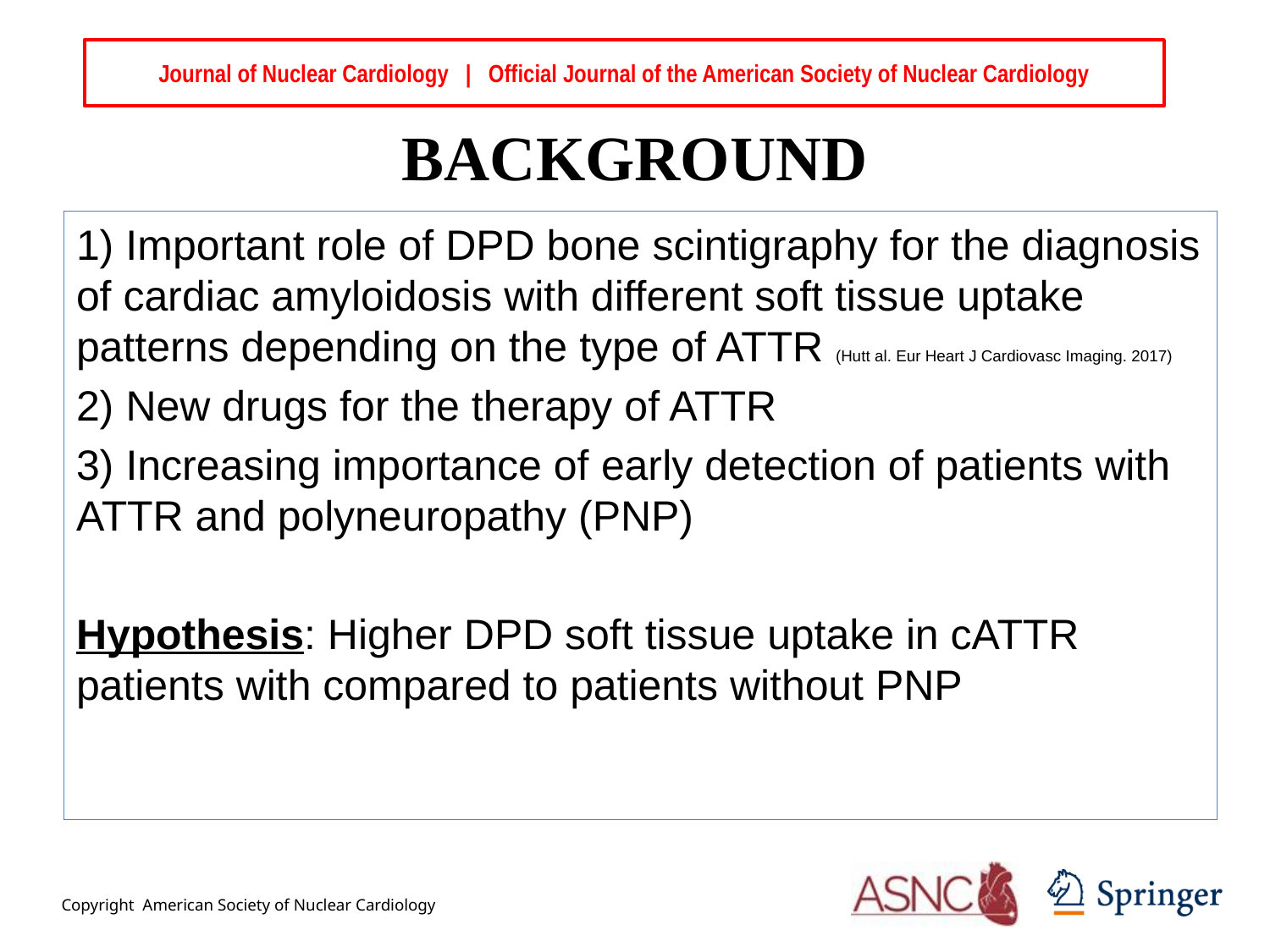

Journal of Nuclear Cardiology | Official Journal of the American Society of Nuclear Cardiology
# BACKGROUND
1) Important role of DPD bone scintigraphy for the diagnosis of cardiac amyloidosis with different soft tissue uptake patterns depending on the type of ATTR (Hutt al. Eur Heart J Cardiovasc Imaging. 2017)
2) New drugs for the therapy of ATTR
3) Increasing importance of early detection of patients with ATTR and polyneuropathy (PNP)
Hypothesis: Higher DPD soft tissue uptake in cATTR patients with compared to patients without PNP
Copyright American Society of Nuclear Cardiology

## Slide 3
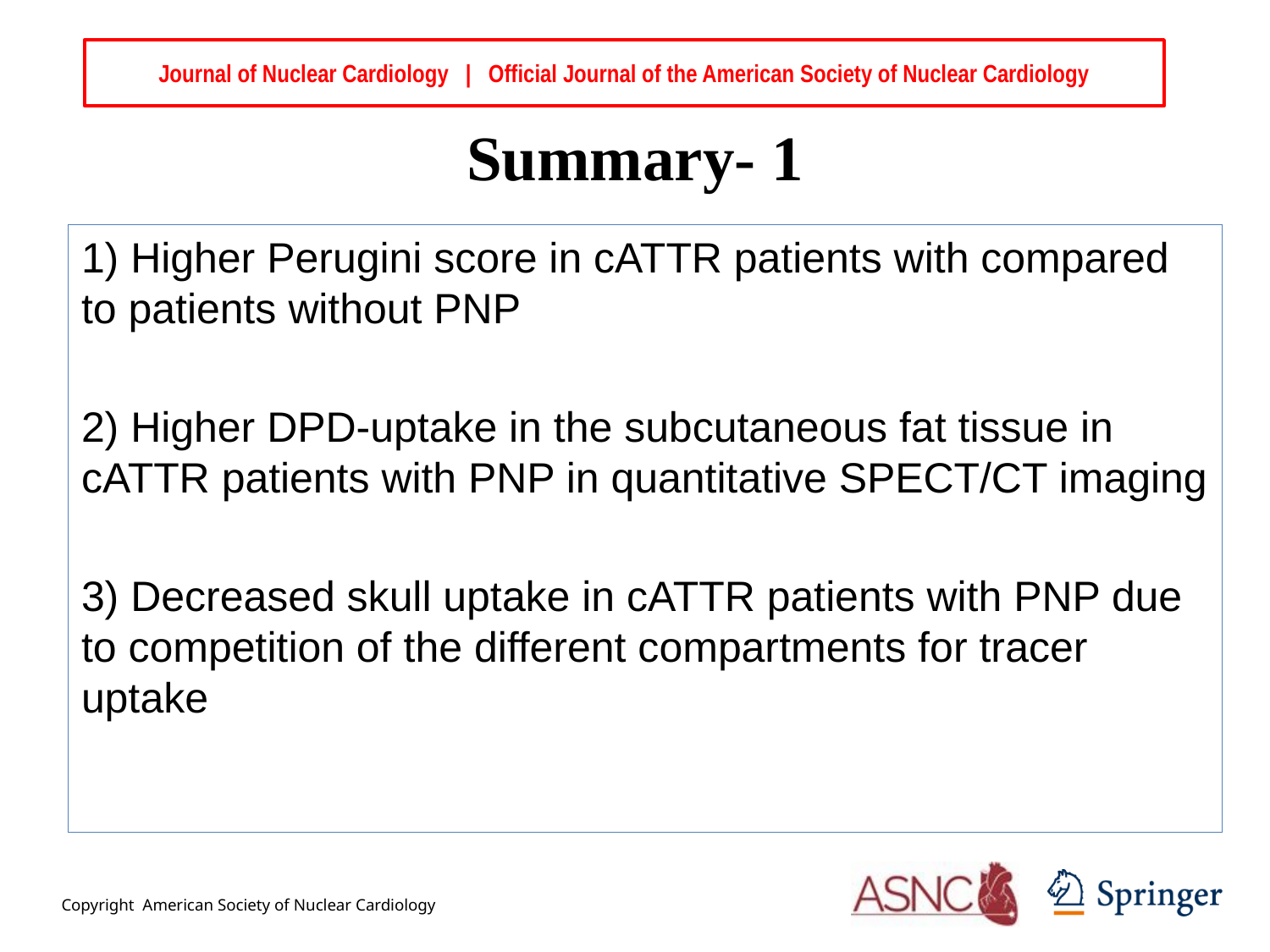

Journal of Nuclear Cardiology | Official Journal of the American Society of Nuclear Cardiology
# Summary- 1
1) Higher Perugini score in cATTR patients with compared to patients without PNP
2) Higher DPD-uptake in the subcutaneous fat tissue in cATTR patients with PNP in quantitative SPECT/CT imaging
3) Decreased skull uptake in cATTR patients with PNP due to competition of the different compartments for tracer uptake
Copyright American Society of Nuclear Cardiology

## Slide 4
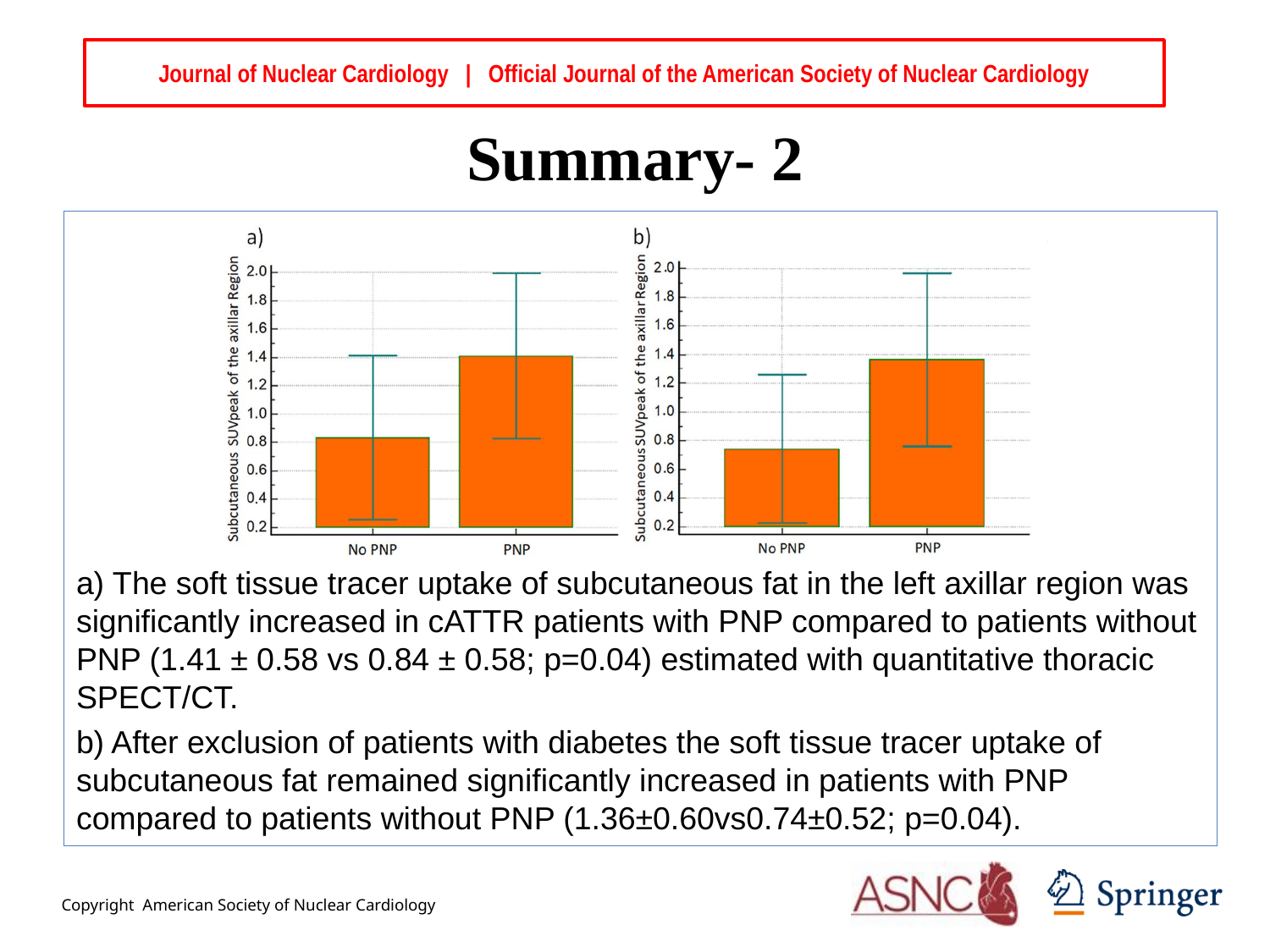

Journal of Nuclear Cardiology | Official Journal of the American Society of Nuclear Cardiology
# Summary- 2
a) The soft tissue tracer uptake of subcutaneous fat in the left axillar region was significantly increased in cATTR patients with PNP compared to patients without PNP (1.41 ± 0.58 vs 0.84 ± 0.58; p=0.04) estimated with quantitative thoracic SPECT/CT.
b) After exclusion of patients with diabetes the soft tissue tracer uptake of subcutaneous fat remained significantly increased in patients with PNP compared to patients without PNP (1.36±0.60vs0.74±0.52; p=0.04).
Copyright American Society of Nuclear Cardiology

## Slide 5
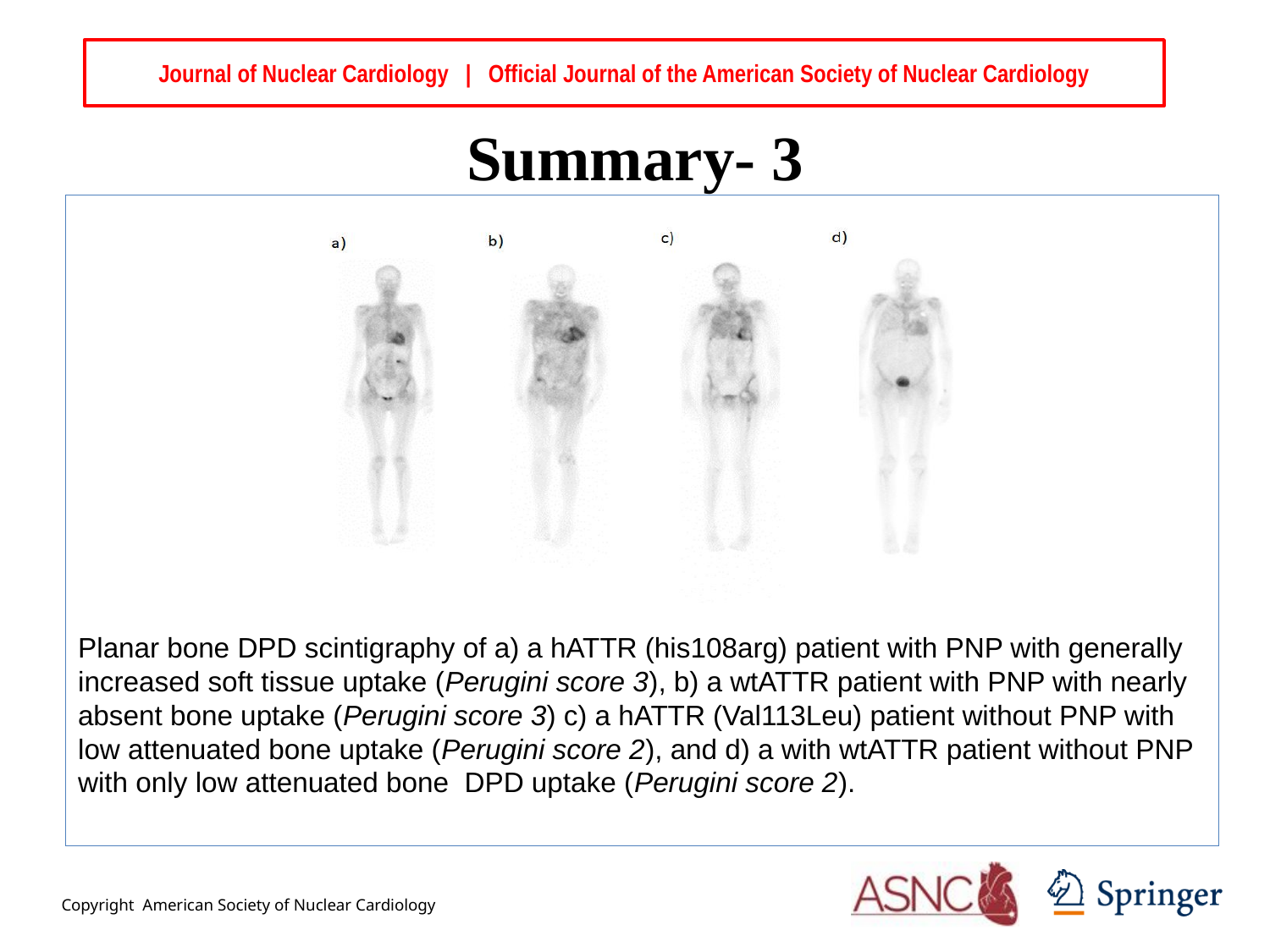

Journal of Nuclear Cardiology | Official Journal of the American Society of Nuclear Cardiology
# Summary- 3
Planar bone DPD scintigraphy of a) a hATTR (his108arg) patient with PNP with generally increased soft tissue uptake (Perugini score 3), b) a wtATTR patient with PNP with nearly absent bone uptake (Perugini score 3) c) a hATTR (Val113Leu) patient without PNP with low attenuated bone uptake (Perugini score 2), and d) a with wtATTR patient without PNP with only low attenuated bone DPD uptake (Perugini score 2).
Copyright American Society of Nuclear Cardiology

## Slide 6
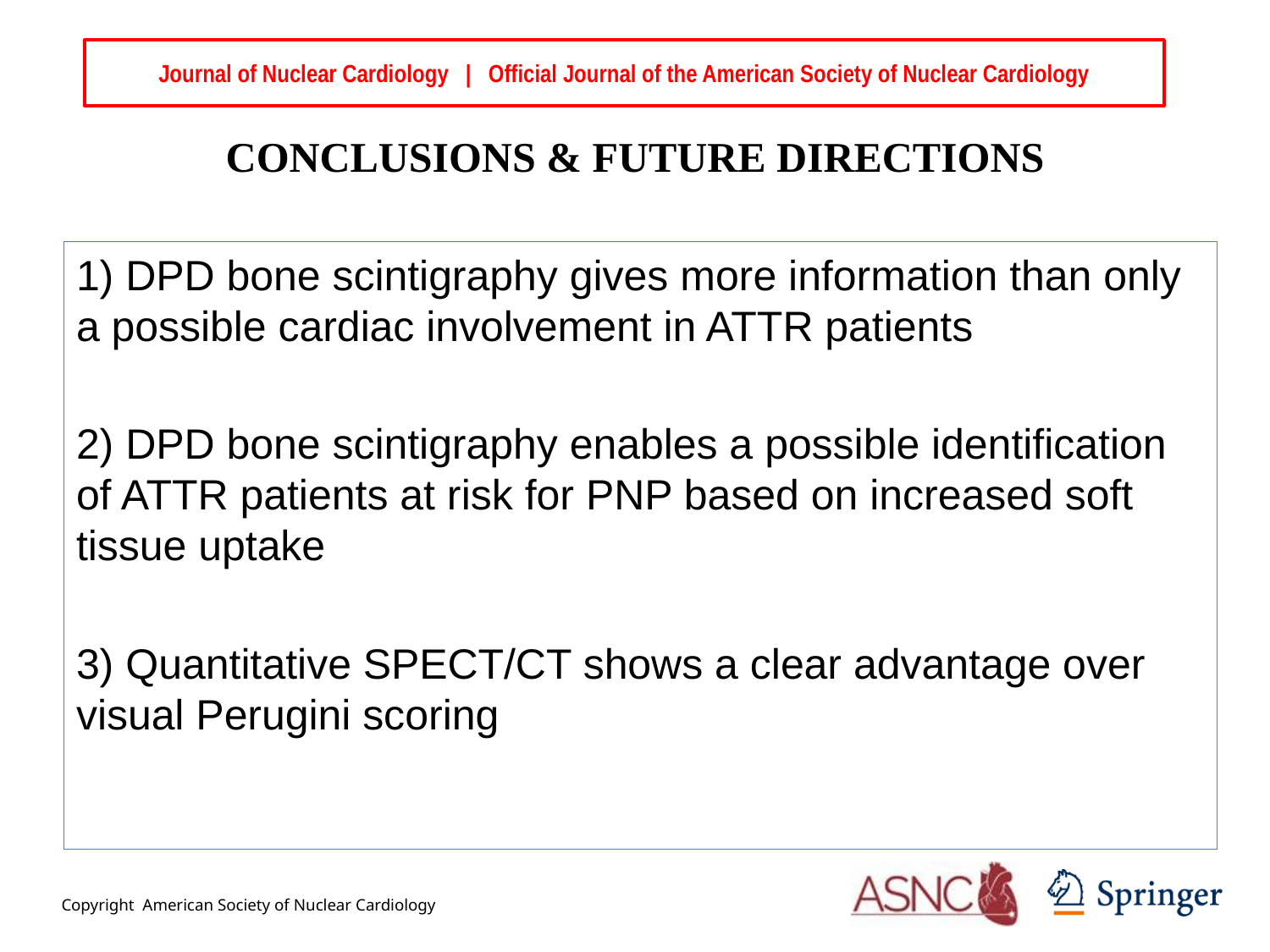

Journal of Nuclear Cardiology | Official Journal of the American Society of Nuclear Cardiology
# CONCLUSIONS & FUTURE DIRECTIONS
1) DPD bone scintigraphy gives more information than only a possible cardiac involvement in ATTR patients
2) DPD bone scintigraphy enables a possible identification of ATTR patients at risk for PNP based on increased soft tissue uptake
3) Quantitative SPECT/CT shows a clear advantage over visual Perugini scoring
Copyright American Society of Nuclear Cardiology
