# Supplementary material for: Does [99mTc]-3,3-diphosphono-1,2-propanodicarboxylic acid (DPD) soft tissue uptake allow the identification of patients with the diagnosis of cardiac transthyretin-related (ATTR) amyloidosis with higher risk for polyneuropathy?
Source: J Nucl Cardiol. 2022 Jul 11;30(1):357–67. doi: 10.1007/s12350-022-02986-7 (PMC9984356; doi:10.1007/s12350-022-02986-7)
Supplement: Supplementary file 2 — Electronic supplementary material 2 (DOCX 12 kb) [file 12350_2022_2986_MOESM2_ESM.docx]

**Fig. ESM3: Representative examples of mATTR and wtATTR patients with and without PNP investigated with DPD SPECT/CT**

a) a patient mATTR (his108arg) and PNP with generally increased soft tissue uptake (Perugini score 3) also affecting the left axillary region, b) a patient with wtATTR and PNP who shows a strongly increased soft tissue uptake c) a patient with mATTR (Val113Leu) without PNP who shows only a slight soft tissue tracer uptake, especially in the region of the femoral shaft and d) a patient with wtATTR without PNP who also shows who also shows slight soft tissue tracer uptake.
