# Supplementary figures and images for: Does [99mTc]-3,3-diphosphono-1,2-propanodicarboxylic acid (DPD) soft tissue uptake allow the identification of patients with the diagnosis of cardiac transthyretin-related (ATTR) amyloidosis with higher risk for polyneuropathy?
Source: J Nucl Cardiol. 2022 Jul 11;30(1):357–67. doi: 10.1007/s12350-022-02986-7 (PMC9984356; doi:10.1007/s12350-022-02986-7)

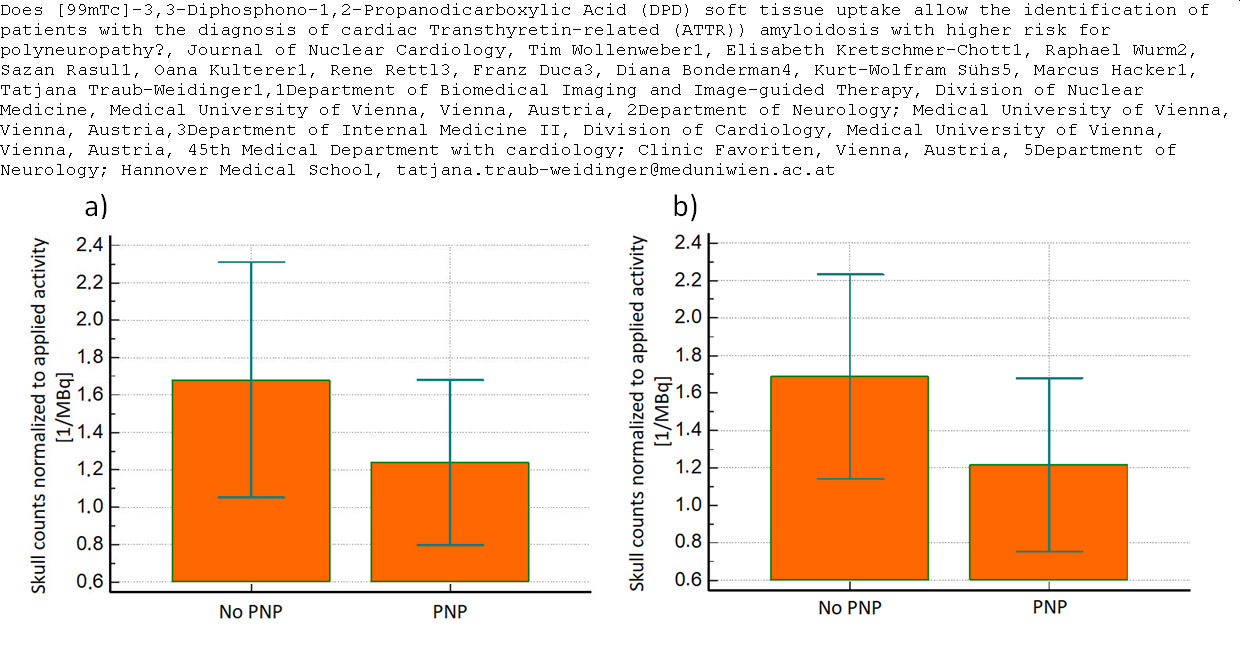

Supplement: Supplementary file 3 — Electronic supplementary material 3 (TIF 432 kb) [file 12350_2022_2986_MOESM3_ESM.tif]

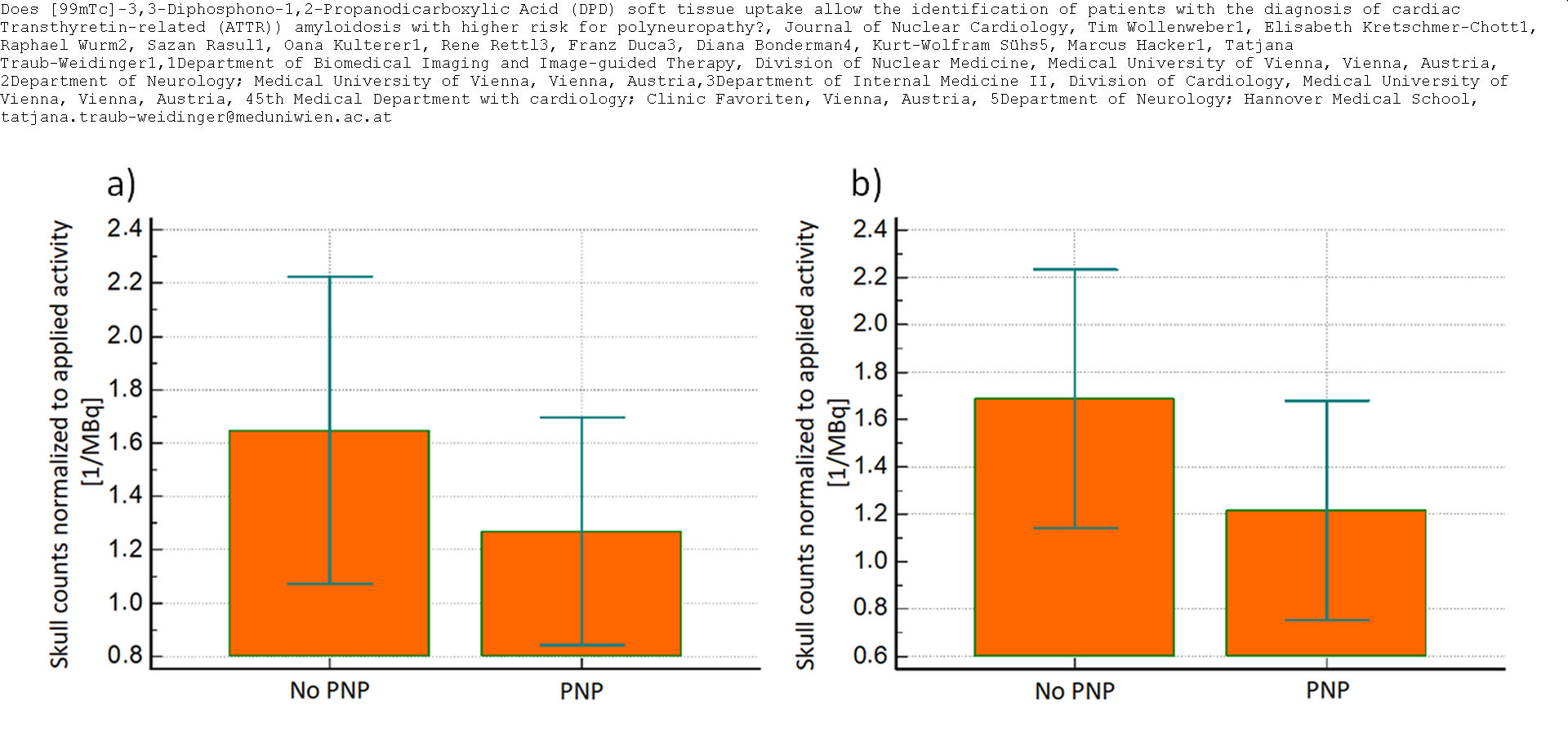

Supplement: Supplementary file 5 — Electronic supplementary material 5 (TIF 568 kb) [file 12350_2022_2986_MOESM5_ESM.tif]

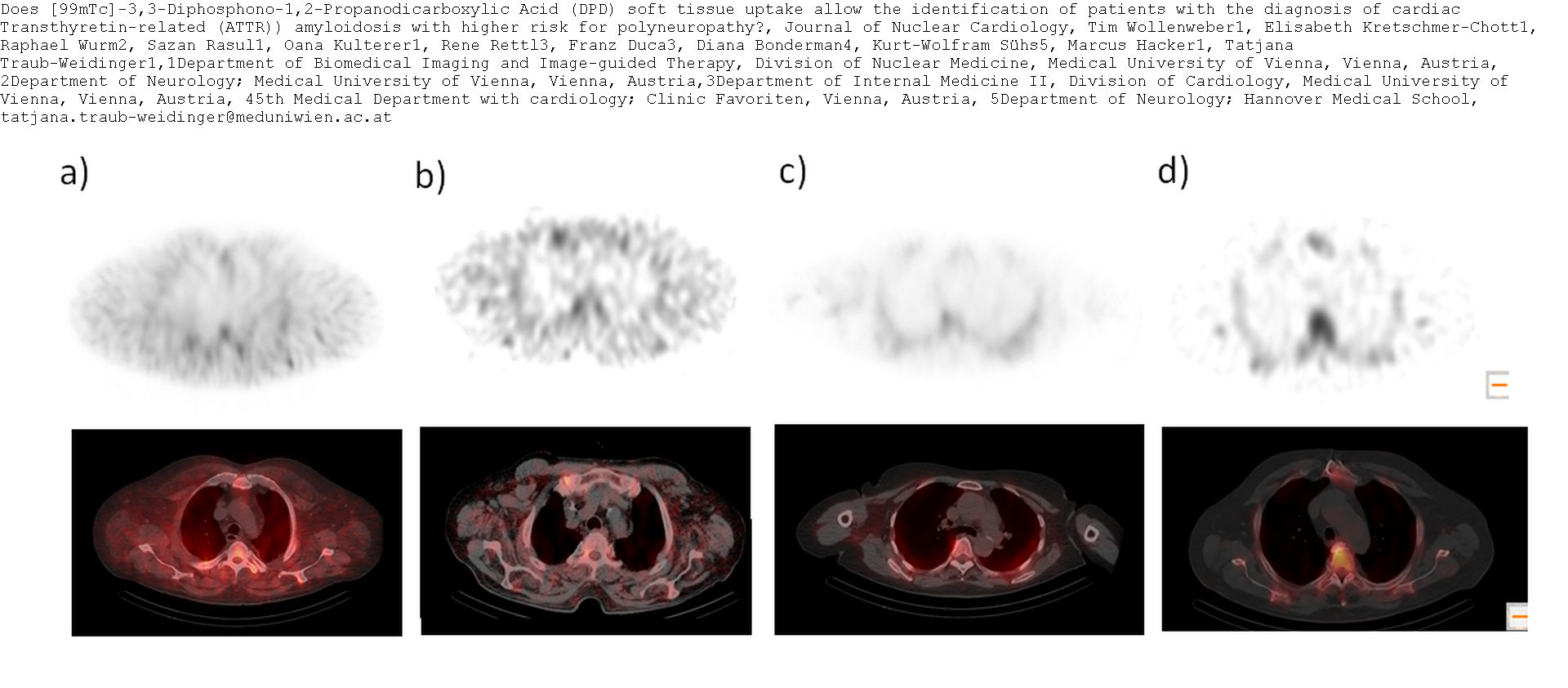

Supplement: Supplementary file 7 — Electronic supplementary material 7 (TIF 544 kb) [file 12350_2022_2986_MOESM7_ESM.tif]
