# Supplementary material for: Does [99mTc]-3,3-diphosphono-1,2-propanodicarboxylic acid (DPD) soft tissue uptake allow the identification of patients with the diagnosis of cardiac transthyretin-related (ATTR) amyloidosis with higher risk for polyneuropathy?
Source: J Nucl Cardiol. 2022 Jul 11;30(1):357–67. doi: 10.1007/s12350-022-02986-7 (PMC9984356; doi:10.1007/s12350-022-02986-7)
Supplement: Supplementary file 4 — Electronic supplementary material 4 (DOCX 13 kb) [file 12350_2022_2986_MOESM4_ESM.docx]

**Fig. ESM1: Comparison of the skull uptake normalized to the applied activity in patients with vs without PNP using planar DPD bone scintigraphy**

a) In the group of the 50 patients with ATTR the tracer-uptake in the skull normalized to applied activity showed a trend to be significantly decreased in patients with PNP compared to patients without (1.24 ± 0.44 vs 1.68 ± 0.63; p=0.01).

b) After exclusion of patient with diabetes mellitus the tracer-uptake in the skull normalized to applied activity became significantly decreased in patients with PNP compared to patients without (1.20±0.46 vs 1.76±0.62; p=0.01).
