# Supplementary material for: Does [99mTc]-3,3-diphosphono-1,2-propanodicarboxylic acid (DPD) soft tissue uptake allow the identification of patients with the diagnosis of cardiac transthyretin-related (ATTR) amyloidosis with higher risk for polyneuropathy?
Source: J Nucl Cardiol. 2022 Jul 11;30(1):357–67. doi: 10.1007/s12350-022-02986-7 (PMC9984356; doi:10.1007/s12350-022-02986-7)
Supplement: Supplementary file 6 — Electronic supplementary material 6 (DOCX 12 kb) [file 12350_2022_2986_MOESM6_ESM.docx]

**Fig. ESM2: Comparison of the skull uptake normalized to applied activity in wtATTR patients with vs without PNP using planar DPD bone scintigraphy**

a) In the group of the 41 patients with wATTR the tracer uptake in the skull normalized to applied activity was significantly decreased in patients with PNP compared to patients without (1.65 ± 0.43 vs 1.65 ± 0.58; p=0.06).

b) After exclusion of patients with diabetes mellitus the tracer uptake in the skull normalized to applied activity was still significantly decreased in patients with PNP compared to patients without (1.22±0.46 vs 1.69±0.54; p=0.02).
